# Attempt to Silence Genes of the RNAi Pathways of the Root-Knot Nematode, *Meloidogyne incognita* Results in Diverse Responses Including Increase and No Change in Expression of Some Genes

**DOI:** 10.3389/fpls.2020.00328

**Published:** 2020-03-24

**Authors:** Sadia Iqbal, John Fosu-Nyarko, Michael G. K. Jones

**Affiliations:** Plant Biotechnology Research Group, College of Science, Health, Engineering and Education, WA State Agricultural Biotechnology Centre, Murdoch University, Perth, WA, Australia

**Keywords:** *in vitro* RNAi, host-induced gene silencing, *Meloidogyne incognita*, nematode control, root-knot nematodes, RNAi pathway, transgenic plants

## Abstract

Control of plant-parasitic nematodes (PPNs) via host-induced gene silencing (HIGS) involves rational selection of genes and detailed assessment of effects of a possible knockdown on the nematode. Some genes by nature may be very important for the survival of the nematode that knockdown may be resisted. Possible silencing and effects of 20 such genes involved in the RNA interference (RNAi) pathways of *Meloidogyne incognita* were investigated in this study using long double-stranded RNAs (dsRNAs) as triggers. Two of the genes, *ego-1* and *mes-2*, could not be knocked down. Expression of six genes (*xpo-1*, *pash-1*, *xpo-2*, *rha-1*, *ekl-4*, and *csr-1*) were significantly upregulated after RNAi treatment whereas for 12 of the genes, significant knockdown was achieved and with the exception of *mes-2* and *mes-6*, RNAi was accompanied by defective phenotypes in treated nematodes including various degrees of paralysis and abnormal behaviors and movement such as curling, extreme wavy movements, and twitching. These abnormalities resulted in up to 75% reduction in infectivity of a tomato host, the most affected being the J2s previously treated with dsRNA of the *gfl-1* gene. For 10 of the genes, effects of silencing in the J2s persisted as the adult females isolated from galls were under-developed, elongated, and transparent compared to the normal saccate, white adult females. Following RNAi of *ego-1*, *smg-2*, *smg-6*, and *eri-1*, reduced expression and/or the immediate visible effects on the J2s were not permanent as the nematodes infected and developed normally in tomato hosts. Equally intriguing was the results of RNAi of the *mes-2* gene where the insignificant change in gene expression and behavior of treated J2s did not mean the nematodes were not affected as they were less effective in infecting host plants. Attempt to silence *drsh-1*, *mut-7*, *drh-3*, *rha-1*, *pash-1*, and *vig-1* through HIGS led to reduction in nematode infestation by up to 89%. Our results show that genes may respond to RNAi knockdown differently so an exhaustive assessment of target genes as targets for nematode control via RNAi is imperative.

## Introduction

RNA interference (RNAi) of plant-parasitic nematode (PPN) genes was first demonstrated for the cyst nematodes *Heterodera glycines* and *Globodera pallida* following optimization of *in vitro* soaking conditions that made use of neurostimulants to enhance uptake of dsRNA in a buffered solution (Urwin et al., 2002). This breakthrough was soon followed by host-induced gene silencing (HIGS) of *Meloidogyne incognita* and *H. glycines* in a now-common approach where host plants are engineered to produce long hairpin RNAs corresponding to essential nematode genes which are then processed into short interfering RNAs (siRNA) that trigger silencing when nematodes feed on cytoplasmic contents of the transgenic plants (Huang et al., 2006; Steeves et al., 2006; Yadav et al., 2006). Since then, the functions or involvement of particular genes in specific molecular or biological processes of many species of economically important PPNs, mostly of the genera *Meloidogyne*, *Heterodera*, *Globodera*, *Pratylenchus*, *Bursaphelenchus*, and *Radopholus*, have been investigated using these approaches (Chen et al., 2005; Bakhetia et al., 2007; Tan et al., 2013; Antonino de Souza Júnior et al., 2014; Fosu-Nyarko et al., 2016; Qiu et al., 2016; Li et al., 2017). Different lengths and forms of double-stranded RNA (dsRNA) triggers have been employed to achieve gene knockdown in the egg and larval stages of these nematodes; these include short (e.g., 42 nucleotides, nt) and long dsRNAs (>100 nt), and 21 nt siRNAs (Dalzell et al., 2010a, b; Arguel et al., 2012; Nsengimana et al., 2013; Marmonier et al., 2019). The primary aim of these studies has been to investigate the role of the nematode genes in host interaction. In most of these cases if any detectable knockdown affects the biology of the nematode then by extension the strategy could be employed to manage the global economic damage nematodes inflict on crop and horticultural plants which currently exceeds the US$125 estimated 15 years ago (Chitwood, 2003).

Successful use of RNAi to control PPNs requires rational selection of essential genes and a detailed assessment of the effects of a possible knockdown after triggering gene silencing in a target nematode. Currently, because the delivery of dsRNA to PPNs via spraying or any other method is impractical, HIGS is the preferred method for assessing the effectiveness of RNAi as a control strategy. The success of HIGS of PPN genes is due in part to the existence of the RNAi amplification system in plants where gene silencing or its effect(s) is perpetuated following the production of secondary siRNAs generated from templates of the trigger dsRNA. Several other host factors such as low level or absence of T-DNA methylation in transgenic plants allowing high levels of expression of dsRNA (hairpin) of the target gene and processing of transcribed dsRNA into functional siRNAs, may have to be favorable for HIGS of a PPN gene to be successful (Bakhetia et al., 2005; Lilley et al., 2007, 2012; Fosu-Nyarko et al., 2017). A survey of current literature and reviews appears to suggest that almost all genes of PPNs are susceptible to RNAi and that once the appropriate stage of a PPN is treated with dsRNA, there is a guarantee of a measurable gene knockdown and a subsequent negative effect or disruption on the biology of the nematode or its ability to successfully interact with its host. To date, few PPN genes have been reported to be somewhat refractory to RNAi where responses to similar amounts of dsRNA that trigger silencing of other genes under similar conditions include no measurable reduction in transcript abundance, a considerable delay in gene knockdown effect after exposure to dsRNA, a relatively quick recovery of transcript to levels before exposure to dsRNA triggers and no obvious deleterious effect of silencing on the behavior and biology of the target nematode (Bakhetia et al., 2008; Dalzell et al., 2010a, 2011; Danchin et al., 2013; Chi et al., 2016; Shivakumara et al., 2016; Nguyen et al., 2018). PPN genes currently reported as refractory to exogenous RNAi have diverse roles in cellular processes and include nematode effectors and the cytosolic RNase III enzyme dicer of *M. incognita.* Among the many reasons suggested for RNAi recalcitrance are the nature, function, and expression turnover of target genes (Dalzell et al., 2011; Danchin et al., 2013; Tan et al., 2013; Chi et al., 2016; Shivakumara et al., 2016; Nguyen et al., 2018).

Genes involved in the specific processes of the siRNA and microRNA (miRNA) pathways play crucial roles in the regulation of many other essential genes and cellular processes (Rosso et al., 2009; Dalzell et al., 2011; Maule et al., 2011; Iqbal et al., 2016; Fosu-Nyarko et al., 2017). The protein products of most of these genes have multifunctional domains implying they may be involved in many unrelated cellular processes, making them essential to the survival of an organism. Knockdown of any such gene may affect multiple mechanisms and hence may severely impair development and viability of PPNs, or because of their importance to the organism, there may be cellular mechanisms (e.g., homeostasis) that may make these genes recalcitrant to RNAi. The aim of this research was, therefore, to assess if knockdown of 20 genes which play significant roles in the RNAi pathways is possible, and if any, how such disruption will affect the behavior and infectivity of J2s of *M. incognita* and their development to adult females. The expected outcomes include a catalog of RNAi phenotypes of these genes which may be important in the future for the development of RNAi mutants for genomics studies and for understanding the mechanism of RNAi of *M. incognita* and PPNs, of which very little is known at present.

## Materials and Methods

### Target dsRNAs and Induction of RNAi via Soaking

Gene products of the 20 genes used in this study have previously been classified into seven functional groups based on their roles in the siRNA or miRNA silencing pathways as RISC proteins, amplification proteins, RNAi inhibitors, transport proteins, dicer complexes, nuclear RNAi proteins, or argonautes (Rosso et al., 2009; Iqbal et al., 2016). The gene sequences used were those identified from genomic contigs of *M. incognita* by Iqbal et al. (2016) and the accession numbers are provided in Supplementary Table S1. Target dsRNAs were generated from amplicons corresponding to coding regions of functional protein domains of the genes and are designated as “dsgene” throughout the manuscript. The sizes of the target genes used to generate dsRNAs ranged from 131 to 696 bp (Supplementary Table S1). The amplicons were obtained from cDNA generated from total RNA of mixed stages of *M. incognita* as described by Iqbal et al. (2016). They were then ligated and cloned using the transcription vector pDoubler, which facilitates transcription with the T7 RNA polymerase (Fosu-Nyarko et al., 2016). Target dsRNAs were synthesized using the HiScribe T7 *In vitro* Transcription Kit (New England Biolabs, Australia) as described by Iqbal et al. (2016) from templates prepared from the cloned amplicons. Also, dsRNA corresponding to two nematode genes with known RNAi phenotypes, *pat-10* and *rol-6*, together with that for the non-target Green fluorescent protein (*gfp*) gene of *Aequorea victoria* were included as controls (Cox et al., 1980; Nsengimana et al., 2013). RNAi of each nematode target gene and the *gfp* control was induced by soaking a total of 7000 freshly hatched J2s of *M. incognita* in three separate biological replicates in M9 buffered solutions each containing 1 mg mL^–1^ of dsRNA with 0.05% gelatine, 50 mM octopamine, and 3 mM spermidine as described by Tan et al. (2013). Also, the behavior and activity of J2s soaked in the buffered solution without dsRNA were used as controls to enable accurate assessment of the effect of dsRNA soaking on the nematodes. To demonstrate uptake of the soaking solution, 1000 J2s were soaked with 1 mg mL^–1^ Fluorescein Isothiocyanate (FITC) and 1 mg mL^–1^ dsRNA of *gfp* (dsgfp); the presence of the FITC in the stylet, gut, and the body of the nematode was used as a sign of uptake of the solution, more likely through ingestion.

### Observation of Nematode Behavior and Infectivity After RNAi Induction

After 16 h incubation, nematodes were removed from the soaking solution, and the integrity of the dsRNA (20 μL) assessed on a 1% agarose gel. The activity and behavior of the nematodes from the three biological replicates (300 J2s per replicate) for each dsRNA and the no-dsRNA controls were observed with an Olympus BX-51 microscope under bright-field, whereas those soaked with FITC were assessed using the FITC filter (450–480 nm) on the microscope. Deviation from regular nematode activity was used to assess RNAi effects in treated J2s. Regular nematode activity was defined based on the behavior of nematodes soaked in buffers without dsRNA, and included movement in a solution typified by “undulatory propulsion in which a train of dorso-ventral waves is passed from the head to the tail” (Wallace, 1968), their sharp reaction to light and posture that indicates they are alive. RNAi phenotypes exhibited by the J2s were described using similar terminology used for *Caenorhabditis* species^1^. Longer term effect of gene silencing on the nematodes was studied by assessing their ability to establish and develop in 2-week-old susceptible tomato seedlings (cv. Grosse Lisse) grown and maintained in white sand in 5 × 4 trays each with a capacity of 120 cm^3^. For each dsRNA and no-dsRNA treatments, 10 plants were each inoculated with 400 J2s and grown under glasshouse conditions (25 ± 5°C) for four weeks. Nematode infectivity was assessed using the number of galls induced on roots per gram of dry root weight of seven of the inoculated plants. Development of treated nematodes to adult females was assessed by dissecting galls of the three remaining plants after a further three weeks; the sizes and structures of the females developed from dsRNA-treated nematodes were compared to those used as controls.

### Assessment of Target Gene Knockdown 16 h After Soaking in dsRNA

Relative abundance of target transcripts after RNAi treatment was assessed using cDNA synthesized from 100 ng of RNA extracted from 2000 J2s, representing each of the three biological replicates of the treatments. Replicates of quantitative PCRs were done with a 1:10 dilution of the cDNA in 20 μL using 10 μM each of primer pairs with the SensiFAST^TM^ SYBR^®^ No-ROX Kit (Bioline Australia). The cycling conditions were an initial denaturation for 10 min at 95°C followed by 40 cycles of 95°C for 15 s, 55°C for 20 s, and 72°C for 30 s in a Qiagen Rotor-Gene Q. Primers used were selected based on their specificities as determined by melting curves, and except for the *M. incognita* actin gene (Genbank: BE225475.1) used for normalizing gene expression, did not bind to sequences of the cDNA region used to generate target dsRNA (Supplementary Table S2). Fold changes in target gene expression were calculated using the comparative Ct method (ΔΔCt); the values obtained represented a mean of the three biological replicates of the soaking experiments (Livak and Schmittgen, 2001).

### Generation of Transgenic Plants for HIGS

Host-induced gene silencing of six of the 20 RNAi pathway genes representative of the broad effects silencing produced on the nematodes were studied further; the aim was to find out if plant-processed dsRNAs and/or siRNAs affected nematode infectivity and development in a host plant in a similar manner as synthesized dsRNA *in vitro*. The selected genes were *vig-1*, *mut-7*, *drsh-1*, *pash-1*, *rha-1*, and *drh-3*. For the gene *drh-3*, the effect of RNAi after soaking has previously been reported by Iqbal et al. (2016). The same target sequences used for the *in vitro* RNAi experiments were used to create hairpin vectors for *Arabidopsis thaliana* transformation. The target sequences were first analyzed with the software “dsCheck” (Naito et al., 2005) using all available public sequences of *A. thaliana* to ensure potential nematode-derived dsRNAs or siRNAs do not have off-target effects on transgenic plants.

Hairpin vector constructs were made by assembling the sense and antisense sequences of target genes from the transcription vector pDoubler to the vector pCleaver (Supplementary Method S1 and Supplementary Figures S1, S2). The hairpin cassette was then ligated to the *Not*I site of the binary vector pART27 (Wesley et al., 2001). Competent *Agrobacterium tumefaciens* strain GV3101 modified with the binary vectors were used to transform *A. thaliana* ecotype Columbia-0 using the floral dip method (Clough and Bent, 1998). Transgenic (T1 and T2) seeds were sterilized with 3% bleach, washed with sterile water, and selected on MS media plates (2.2 g L^–1^ Murashige and Skoog basal medium with Gamborg vitamins, 3% sucrose, 0.8% Agar) supplemented with 50 mg L^–1^ of kanamycin monosulphate for two weeks. Surviving transgenic seeds were transferred to soil in 200 mL plastic cups in a growth chamber (Conviron ATC40, 16-h light @150 μmol m^–2^ s^–1^, 40% RH, and 23 ± 2°C). Seeds were collected using “Aracons” fitted with transparent plastic tubes^2^. T-DNA integration into the T2 seedlings was confirmed by amplifying 364 bp of the *nptII* gene, which confers resistance to Kanamycin using the primer pair NptII-F and NptII-R (Supplementary Table S1) in a standard 20 μL reaction from 200 ng of genomic DNA extracted from leaves using the CTAB method (Iqbal, 2015). Transcription of the nematode sequence (the hairpins) from transgenic T2 seedlings was assessed by reverse transcription-PCR using cDNA synthesized from 500 ng of RNA isolated from leaves. The total RNA extraction and cDNA synthesis procedures were as described for nematodes. PCRs with the template cDNAs were conducted with nematode gene-specific primers (Supplementary Table S1) using a 1:10 dilution of cDNA.

### Nematode Challenge and Assessment of Infection

Effects of HIGS on *M. incognita* were assessed using establishment and development of infective J2s in 3-week-old T2 transgenic Arabidopsis plants. Fourteen replicate plants for each transgenic line harboring a hairpin of a nematode target gene, or the non-target *gfp* gene and 20 replicates of wild-type plants were each inoculated with 200 freshly hatched J2s. Inoculation was carried out by placing the nematodes suspended in water up to 2 cm deep in four places around the roots. Inoculated plants were maintained for four weeks after which 10 replicates per transgenic line and 16 of the wild-type plants were examined; the number of galls on infected roots was used as a measure of nematode infectivity. The development of J2s to adult nematode females in the infected plants was also assessed by dissecting the latter out of the remaining replicate plants a week later. The females were then stained with Acid Fuchsin (Byrd et al., 1983), and observed with an Olympus BX-51 microscope, comparing their morphological/structural features with nematodes that developed on wild-type plants.

### Statistical Analysis

The SPSSv20 software package (IBM Corporation, United States) was used for the analysis of variance (ANOVA) and calculations of means, standard deviation, and standard error of the mean number of galls per gram of dry root of nematode-infected tomato (after dsRNA soaking experiments) and transgenic Arabidopsis plants. Significance between treatments was tested at *p* < *0.05*, and pair-wise comparisons were done *post hoc* using the Tukey’s test. Student’s *T*-test in Microsoft Excel Analysis ToolPak was used to establish significant differences (*p* < *0.05*) between target gene expression and to construct bar charts with error bars.

## Results

### Ingestion of dsRNA Mostly Induced Behavioral Changes in *M. incognita* J2s

**FIGURE 1:**
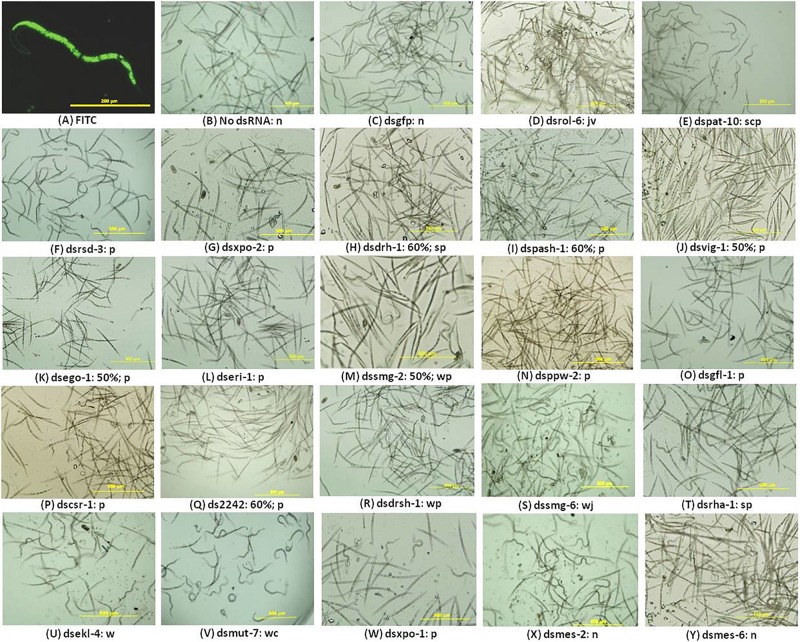
RNAi phenotypes of *M. incognita* J2s after soaking in buffered solution containing dsRNA (1 mg mL^–1^) of 22 nematode genes. **(A–E)** Control RNAi treatments with J2s soaked with 1 mg mL^–1^ FITC **(A)** or soaked without dsRNA **(B)** or with dsRNAs of the *gfp* gene **(C)**, or of the nematode genes *rol-6* **(D)**, and *pat-10* **(E)**. **(F–Y)** J2s treated with dsRNA of 20 genes of the RNAi pathway. Images are denoted by the dsRNA (ds) of the target gene, percentage of nematodes affected after RNAi, and the RNAi phenotypes denoted as n = normal, s = straight, p = paralyzed, c = curvy, w = wavy, j = captured during a jerky action, v = captured during vigorous movement. No percentage is indicated where over 60% of the nematodes exhibited one or more of the phenotypes indicated. Scale bar: 200 μm (FITC) and 500 μm. Phenotypic observations and estimates of percentages of nematodes exhibiting any phenotype were based on three biological replicates.

The presence of FITC in the stylet, gut, and body of the nematodes soaked in the M9 buffer with the dye was an indication that the worms ingested some of the solution implying the J2s would have ingested dsRNA where it was included in treatments (Figure 1A). It could not be ruled out that some of the solution may have entered the nematode body through other orifices. Gel electrophoresis analyses of the integrity of the dsRNA in the buffered solutions before and after nematode soaking indicated there was no significant degradation during the 16-h incubation period (data not shown). It can, therefore, be inferred that the nematodes ingested intact dsRNA during the incubation period. Treated nematodes were observed under the microscope for 30 min during which time their posture, movement, and attraction to light were observed and documented in real time. For each treatment, the percentages of the nematodes exhibiting phenotypic features different from the controls were also computed. The behavior and phenotypes of some nematodes changed over time; that is, they displayed more than one phenotype. Nematodes which exhibited a consistent phenotype during the observation were typically dead, inactive, or paralyzed. Figure 1 is a composite still image showing some of the observed phenotypes exhibited by treated nematodes; the most prominent phenotypes and the percentage of nematodes displaying phenotypes are described in the legend and the text. A small percentage (<5%) of the nematodes soaked without dsRNA or with dsgfp were inactive or showed limited movement on exposure to light (Figures 1B,C). The rest were very active and reacted very quickly to light from a microscope with a typical sinusoidal wave motion away from the source of light. This is typified by the curvy posture (where a nematode is curled to a “c” or “l” shape) or wavy posture signifying a moving nematode. In contrast, J2s soaked in dsrol-6 displayed vigorous involuntary movements, a behavior akin to twitching (Figure 1D). Nematodes soaked with dspat-10 were mostly paralyzed; this phenotype was used to refer to nematodes where there was no movement in any part of the body or minimal movement in the head region for over 10 min on exposure to light (Figure 1E). The posture of the paralyzed nematodes varied; some were straight and did not move at all when exposed to light whereas others were curved in a position similar to the controls but their bodies were in fact rigid in that position for long periods of time indicating they had probably suddenly stopped movement (Figure 1E).

The posture and behavior of nematodes soaked in dsRNAs of 18 of the 20 target genes suggested they were affected by the treatments (Figures 1F–W). Based on all the RNAi phenotypes observed for each treatment, the nematodes were broadly put into three groups: the first consisted of nematodes treated with dsRNAs targeting 12 of the genes, namely, *rsd-3*, *xpo-2*, *drh-1*, *pash-1*, *vig-1*, *ego-1*, *eri-1*, *smg-2*, *ppw-2*, *gfl-1*, *csr-1*, and *2242* with various degrees of paralysis (Figures 1F–Q). The affected J2s barely moved when exposed to light and remained straight and unresponsive with movement restricted to the head region. Between 10 and 60% of the J2s treated with dsRNAs of *drh-1*, *pash-1*, *2242*, *ego-1* and *vig-1* appeared dead as they were straight and did not respond to light or show any form of movement. Generally, the level of paralysis of the RNAi-affected nematodes was differentiated by the posture of the nematodes over the period of examination after the 16-h long dsRNA treatment; while some paralyzed nematodes were straight, others were curled to a “c” or “l” shapes or were occasionally paralyzed in a wavy position. The second group consisted of J2s soaked with dsRNAs of *drsh-1*, *smg-6*, *rha-1*, and *ekl-4*; they maintained normal curvy and wavy nematode posture but were slower in reacting to light, except for those treated with dsRNA of *smg-6* where ∼60% were observed with infrequent jerky movements in the body (Figures 1R–U). The third group consisted of J2s with extreme curling of the whole body with little movement and this was observed mainly for nematodes soaked with dsmut-7 and to a lesser extent those treated with dsxpo-1 (Figures 1V,W). The phenotypes of nematodes treated with dsRNAs of *mes-2* and *mes-6* were typical of the controls; their posture, response to light, and movement were similar to those treated without dsRNA or with dsgfp (Figures 1X,Y).

### Transcript Changes in RNA-Induced Nematodes

**FIGURE 2:**
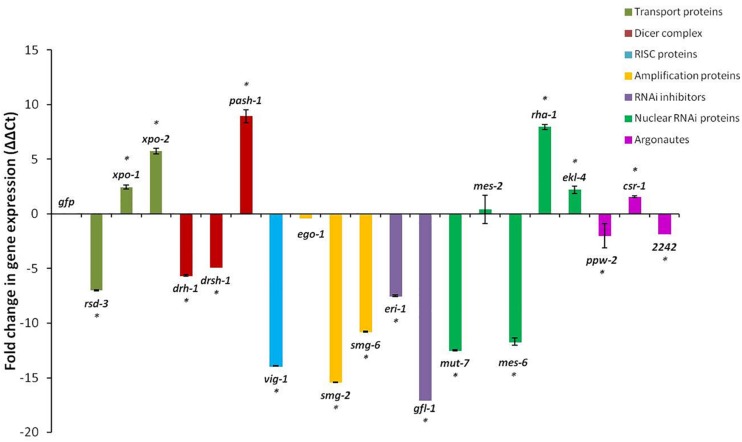
Fold change in target gene expression of *M. incognita* J2s 16 h after soaking in dsRNA of target genes. Expression in nematodes soaked in dsgfp was used as the baseline. Different colored bars represent different groups of genes involved in the small RNA pathway of the nematode. Bars represent mean fold change in expression with standard error of the means representing three biological replicates (significance ^∗^*p* < 0.05).

Changes in the expressions of the 20 genes in the J2s 16 h after RNAi treatment, compared to those in J2s treated with dsRNA of the *gfp* gene, were not consistent. Although generally, RNAi treatment of J2s is expected to result in down-regulation of target genes, expression of just 12 of the 20 genes were significantly reduced with up to 17-fold reduction in expression for the *gfl-1* gene and 15- and 14-fold reductions for the *smg-2* and *vig-1* genes, respectively (*p* < 0.05, Figure 2). There was no significant (*p* < 0.05) change in expressions of the *ego-1* and *mes-2* genes after dsRNA treatment. For the two exportins (*xpo-1*, *xpo-2*), the helicase interactor (*pash-1*), the nuclear RNAi genes (*rha-1*, *ekl-4*), and the argonaute, *csr-1*, there was generally a significant two to nine-fold increase in gene expression in the J2s treated with the respective dsRNAs of the target genes (Figure 2).

### Effect of RNAi on Infectivity and Development of J2s

**FIGURE 3:**
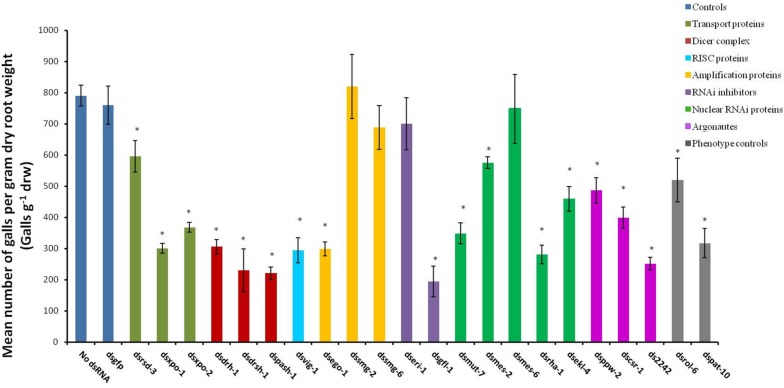
Mean number of galls per gram of dry root of tomato plants inoculated with *M. incognita* J2s 16 h after the nematodes were soaked in dsRNA of target genes. Dsgfp and no dsRNA treatments were used as controls. Different colored bars represent different groups of genes involved in the small RNA pathway of the nematode. Bars represent means (*n* = 7) ± standard error with significant differences between the means and those of the no-dsRNA controls indicated with (^∗^*p* < 0.05).

The abnormal phenotypes induced by RNAi of 16 of the target genes persisted and evidently affected the treated nematodes’ ability to infect host plants. This effect was reflected as significant reduction in the number of galls per gram of dry root weight (galls g^–1^ drw) induced on the tomato host (*p* < 0.05, Figure 3) compared to those on roots inoculated with the control J2s (those treated in buffers with no dsRNA or with dsgfp). The paralyzed J2s treated with dspat-10 and dsrol-6 respectively induced 34% and 60% fewer galls (Figure 3). The most significant reduction in infectivity was observed for nematodes treated with dsgfl-1 (75%, Figure 3). RNAi of 10 other genes, including the two genes of the miRNA processor complex (*drsh-1* and *pash-1*), resulted in 50% or more reduction in infectivity. Infectivity of J2s treated with dsrsd-3 and dsmes-2 was 25% less than those of the control nematodes. RNAi of *mes-6*, *smg-2*, *smg-6*, and *eri-1* did not significantly reduce nematode infectivity of the host tomato (*p < 0.05*) although, except for nematodes treated with dsmes-6, there were visible phenotypic effects on J2s treated with dsRNA of all the genes.

**FIGURE 4:**
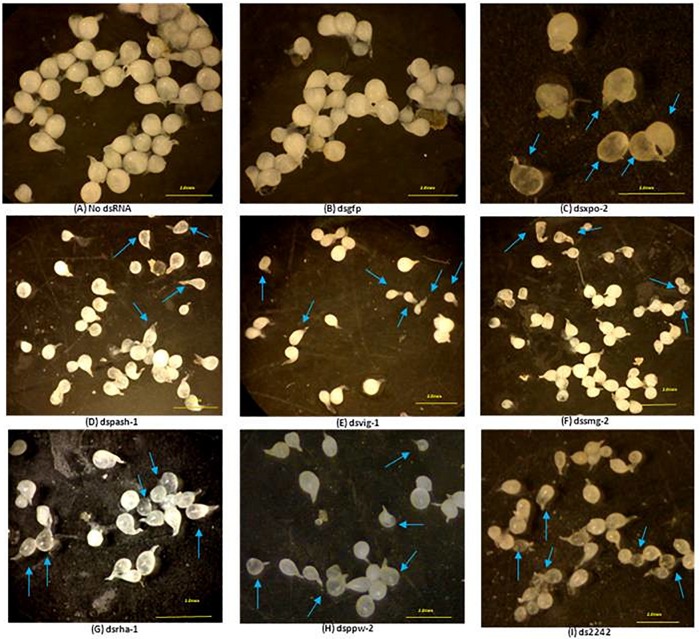
Most significantly affected *M. incognita* females dissected from tomato roots seven weeks after inoculation with treated J2s. Morphology of females derived from J2s treated without dsRNA **(A)**, with dsRNA of the *gfp* gene **(B)** and with dsRNAs of the nematode genes *xpo-2* **(C)**, *pash-1* **(D)**, *vig-1* **(E)**, smg-2 **(F)**, *rha-1* **(G)**, *ppw-2* **(H)**, and *2242* **(I)**. Comparisons were made with females isolated from roots treated with no dsRNA and dsRNA of the *gfp* gene. Arrows point to malformed or underdeveloped females in different treatments. Scale bar: 2 mm.

Development of some dsRNA-treated J2s in tomato roots was also affected. For example, 42% of adult females that developed from dsmut-7-treated J2s had elongated bodies (fusiform) whereas 30% of adult females of dsvig-1-treated J2 lineage were generally smaller. Also, adult females of the J2 lineage treated with dsRNAs of *xpo-2*, *rha-1*, *2242*, *and pash-1* were mostly translucent compared to the pearly white appearance of the females that developed from the control J2s (Figure 4). Whereas 100% of adult females that developed from dsmut-2 J2s were generally smaller and/or translucent, this percentage was 50% for dsxpo-2 treated J2s, about 75% for dsrha-1-treated J2s and 30% for ds2242-treated J2s. Although the J2s treated with dssmg-2 exhibited a wavy or paralyzed phenotype, the number of galls they induced on tomato plants was similar to those for the control nematodes (*p < 0.05*). It was, however, clear that their development was affected as the adult females isolated from infected plants were generally smaller, and some were under-developed and translucent (Figure 4). This observation was similar to adult females of dsppw-2-treated J2s, which also had significantly reduced infectivity. No association could be established between the RNAi phenotypes exhibited by J2s after dsRNA soaking, their subsequent infectivity on tomato plants and morphology of developed adult females.

### Transgenic Arabidopsis Expressing Hairpin of Target Genes

T2 transgenic Arabidopsis plants carrying hairpin of six of the target genes for which silencing via dsRNA soaking reduced nematode infectivity were used for HIGS. Varying numbers of Kanamycin-resistant and PCR-positive (for the *nptII*) T1 lines were generated: 10 for *drh-3*, eight each for *vig-1* and *mut-7*, 12 for *drsh-1*, six for *pash-1*, 18 for *rha-1*, and 13 lines for the *gfp* gene, which were used as the transgenic controls. From these, T2 lines were generated and used for nematode bioassays; they had the expected segregation ratios and no developmental defects as their physical characteristics (e.g., plant height, leaf sizes and shape, flower size, and flowering time) were similar to those of wild-type Col 0 ecotype and the Gfp lines. These were 10 lines each for *drh-3*, *rha-1*, and *drsh-1*, five each for *mut-7* and *pash-1*, four for *vig-1*, and nine for the *gfp* gene. RT-PCR was used to confirm expression of the nematode and Gfp hairpins (genes) in the T2 lines as shown in the representative gel image in Supplementary Figure S3. The intensities of the amplicons varied for different transgenic lines, possibly indicative of differences in expression levels of the transgenes. For replicate plants of line pash1-4, although *in vitro* kanamycin screening and PCR of the *nptII* gene indicated the T-DNA was integrated, no transcript of the transgene was detected on agarose gels (Supplementary Figure S3). It was hypothesized that the expression of the transgene could be very low and perhaps undetectable, so the line was included in the nematode bioassays.

### HIGS of Target Genes Affects *M. incognita* Infectivity and Development

**FIGURE 5:**
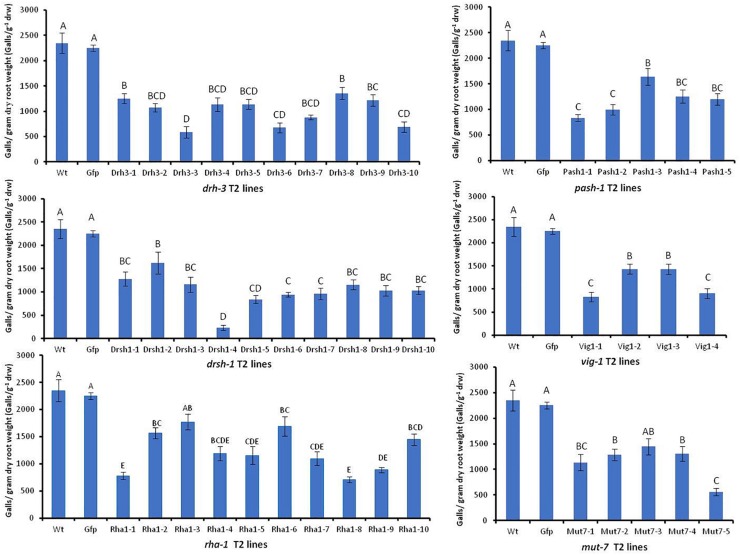
Mean number of galls per gram of dry root weight induced by *M. incognita* J2s on wild-type and transgenic Arabidopsis expressing hairpins of six *M. incognita* genes and the *gfp* gene. Bars represent mean number of galls per gram dry root weight (*n* = 9–16) ± standard error. Significant differences (*p* < *0.05*) are indicated by different letters.

Effects of HIGS were assessed by comparing the mean number of galls g^–1^ drw induced on roots and the sizes, structure, and appearance of females associated with galls of the wild-type Arabidopsis and Gfp lines with those on other transgenic plants four weeks after inoculation. There was no significant difference in the mean number of galls g^–1^ drw induced on the wild-type plants and the replicates of nine Gfp T2 lines (*p* < 0.05, detailed data on Gfp lines are in Supplementary Figure S4). The mean of the galls g^–1^ drw of all Gfp T2 lines was then used for comparison with those of lines expressing target nematode hairpins (Figure 5). Forty-two of the 44 independent lines expressing hairpins of the six target genes, except lines mut7-3 and rha1-3, had a significantly lesser mean number of galls g^–1^ drw than the control wild-type and Gfp lines (*p* < 0.05, Figure 5). The drsh1-4 line had the least followed by line drh3-3 with about 90 and 75% reduction in infectivity, respectively. Overall, the percentage reduction of nematode infection levels by the different lines ranged from 25 to about 90%. Lines of drh-3 reduced infection levels, by 42–75% with seven out of the ten lines reducing infection levels by 50% or more. Eight out of the 10 lines of drsh-1 reduced infection by more than 50%. Forty per cent of lines of pash-1 and mut-1, and 50% of lines of vig-1 and rha-1 reduced the infectivity of nematodes by more than 50% compared to the controls (Figure 5).

**FIGURE 6:**
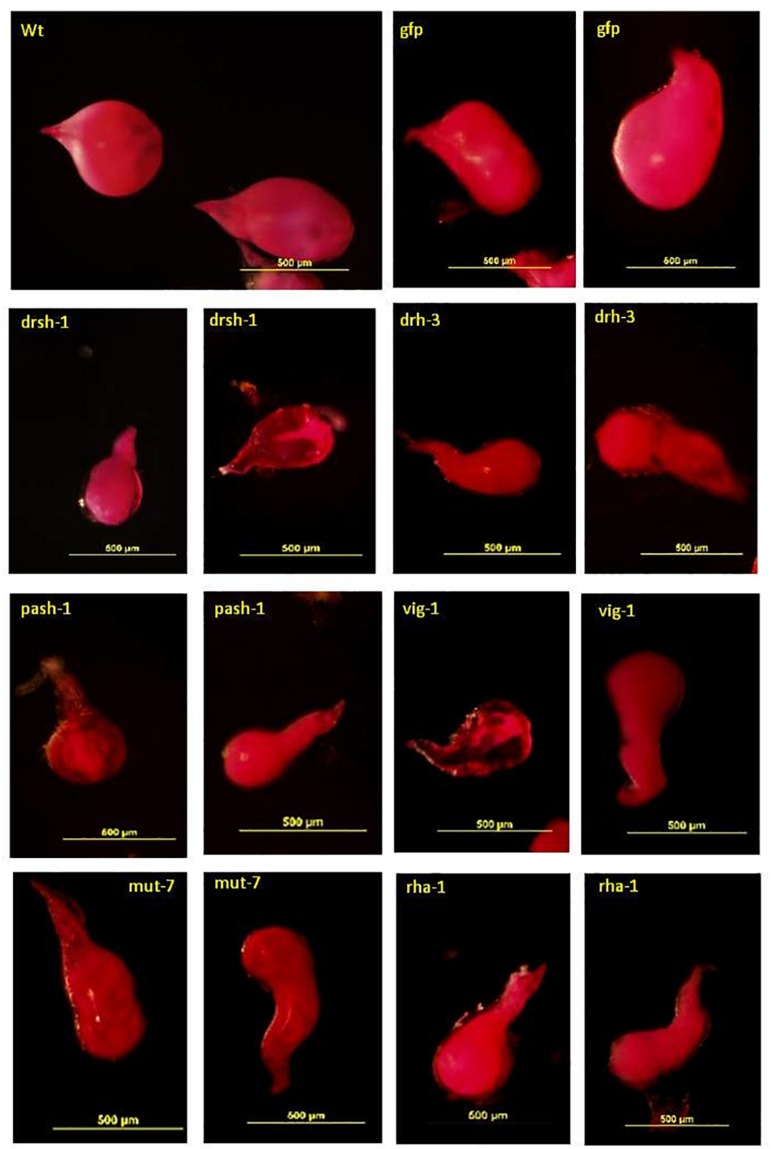
Malformation in Acid Fuchsin-stained *M. incognita* females isolated from transgenic plants expressing hairpins of nematode genes. Scale bar: 500 μm.

Acid Fuchsin staining of the adult females isolated from lines with a significant reduction of infectivity indicated normal development of the J2s was also affected (Figure 6). The deformed females were mostly transparent with a sickly appearance. Those isolated from roots of lines expressing *drh-3*, *drsh-1*, *pash-1*, and *rha-1* were mostly elongated (fusiform), that is, thinner at the posterior end compared to the normal saccate large females in roots of the wild-type plants and the Gfp lines (Figure 6). They made up 65% of females isolated from roots of lines of pash-1, 62% from lines of rha-1, 56% from lines of drh-3, and 46% from lines of drsh-1. About 15–20% of adult females isolated from galls on infected drsh-1, pash-1, and vig-1 lines were similar in shape to those of the controls but were markedly smaller in size, possibly as a result of delayed or impaired development. Abnormalities in some of the females from roots of pash-1 and vig-1 lines manifested as a lack of consistency in the texture and content of the developing female, notably in more than 50% of those isolated from roots of the vig-1 and drsh-1 lines.

## Discussion

In our previous study of the effects of RNAi of six genes with roles in the RNAi pathways, namely, *drh-3*, *tsn-1*, *rrf-1*, *xrn-2*, *mut-2*, and *alg-1*, the behavior and infectivity of the treated J2s of *M. incognita* varied (Iqbal et al., 2016). Notably the infectivity of nematodes treated with dsRNAs targeting the *tsn-1* and *rrf-1* genes were not affected although the phenotypes of the pre-infective J2s suggested they were affected by the dsRNA treatment. We therefore extended this work to 20 other genes known to play key roles in the small and miRNA biosynthesis pathways of *M. incognita*. Most of these genes are also required for developmental regulation so a disruption in the normal functioning of their products will likely induce various forms of stresses that can express as behavioral changes or longer term abnormalities in development. However, because the RNAi system is an essential regulator for many cellular processes, allowing the expression of critical genes to be downregulated may be detrimental to the nematode. This hypothesis was tested by inducing RNAi of 20 of such key genes in *M. incognita* J2s followed by an assessment of target gene knockdown, and observation of the nematode behavior, their infectivity, and development in a tomato host.

This is the first study to report on RNAi of a large number of genes involved in various aspects of the RNAi pathways of any PPN. Besides our previous report (Iqbal et al., 2016), the only other study on RNAi of any of these genes involved the silencing of the drosha, pasha, and dicer genes of juveniles of *M. incognita* using siRNAs (Dalzell et al., 2010b). In the current study, transcript abundance of the 20 genes following soaking of nematodes in dsRNA varied; for *mes-2* and *ego-1*, there was no significant change, for six genes (*xpo-1*, *xpo-2*, *pash-1*, *rha-1*, *ekl-4*, and *csr-1*) there were significant increases whereas there were significant decreases for the rest (12) of the genes. In most reported cases where RNAi of various genes of PPNs was induced following similar conditions as used in this study, there is almost always a case of reduction in gene expression (Tan et al., 2013; Fosu-Nyarko et al., 2017). This report and those of a few genes of *Meloidogyne*, *Heterodera*, and *Globodera* spp. suggest a paradox is now emerging which indicates that all PPN genes are not susceptible to RNAi to the same degree and that there may even be some that are refractory to exogenous RNAi where either no measurable reduction or significant increase in gene expression is observed following exposure of J2s or eggs to long dsRNAs or siRNA triggers (Bakhetia et al., 2008; Dalzell et al., 2010a, 2011; Danchin et al., 2013; Chi et al., 2016; Shivakumara et al., 2016; Nguyen et al., 2018). These genes, which include those for the effectors glutathione S-transferase (*Migst*-1) and venom-allergen-like proteins *Mivap-2*, and the cytosolic RNase III enzyme dicer of *M. incognita* (*Midcr-1*), play different roles in cellular processes, and with the exception of the latter, are not known to be directly involved in the RNAi pathways (Rosso et al., 2009; Dalzell et al., 2011; Maule et al., 2011; Iqbal et al., 2016). Their function may, therefore, not necessarily be the cause of the seeming resistance to exogenous RNAi. Instead, other factors, such as temporal and rate of target gene expression in the J2s of PPNs, the amount of dsRNA used as triggers, the nematodes’ susceptibility to RNAi or its need to maintain homeostasis following an initial change in expression of some genes, may play a part in a nematode’s ability to resist RNAi of a gene or to recover from the effects of silencing particular genes. None of the factors above has so far been directly investigated as the cause of recalcitrance to exogenous RNAi or recovery from the effects of RNAi in nematodes. However, in many reports including those involving RNAi of *Migst*-1, *Mivap-2*, and *Midcr-1*, (Rosso et al., 2009; Dalzell et al., 2011; Maule et al., 2011), suggestion of the nematodes’ need to maintain consistent gene expression levels has strongly been put forward as a possible major factor. For those dsRNA-treated nematodes where there was no immediate reduction in gene expression, delayed depletion of transcripts cannot be ruled out as the reason for the general lack of infectivity of the nematodes in the tomato host. It is possible that increasing the amount of dsRNA triggers in the soaking solutions could have resulted in gene knockdown of some of the recalcitrant genes as has been reported for the *unc-87* gene of the root-lesion nematode, *Pratylenchus thornei* (Tan et al., 2013). However, because RNAi is not known to be either stoichiometric (where a certain level or concentration of dsRNA can be prescribed to cause specific level of gene knockdown) or stochastic (where random events including any amount of dsRNA triggers RNAi), it is difficult to suggest that the amount of the dsRNA trigger used in this study was potentially lower than a threshold required to cause significant reduction in expression of the refractory genes. On the other hand, it can be concluded that for those genes where silencing significantly affected the biology of the nematodes, the amount of the dsRNA used effectively triggered RNAi.

The level of knockdown of the genes we studied could also have been affected by the presence of paralogs. For example, where the dsRNAs used targeted and successfully silenced all functional paralogs of a gene, the expected effect on the nematode could be more significant if the paralogs had the same function. It may, however, not be the case if some paralogs were functionally redundant or have functionally diverged over time. This implies that whether the presence of paralogs could affect the level of silencing of any of the genes we studied can only be empirically or experimentally determined. For these reasons and also the lack of detailed and clearly defined sequences of paralogous genes of PPNs, we did not focus on whether the genes we studied had paralogs or not. Recent advances in genome sequencing technologies have made it possible to study the detailed biology of several species of root-knot nematodes including *M. incognita*, *Meloidogyne hapla*, *Meloidogyne arenaria*, *Meloidogyne floridensis*, *Meloidogyne javanica*, *Meloidogyne enterolobii*, *and Meloidogyne graminicola* (Abad et al., 2008; Opperman et al., 2008; Lunt et al., 2014; Szitenberg et al., 2017; Sato et al., 2018; Somvanshi et al., 2018). However, the presence of paralogs and their possible functions in the nematodes’ genomes are only recently becoming available (Blanc-Mathieu et al., 2017; Pratx et al., 2018). A more recent study of the epigenetic machinery of *Meloidogyne* species has predicted the presence of paralogs of some genes involved in the small RNA pathways (Pratx et al., 2018). For the genes we studied where silencing caused any measurable RNAi effect on the nematode, there is the potential to use them as targets for control of the nematode using RNAi. It is also expected that once the presence of predicted paralogs of genes involved in the small RNAi pathway has been validated using methods such as southern hybridization, the RNAi phenotypes and the effect of silencing reported here would be a useful reference for functional characterization.

For four of the target genes *ego-1*, *smg-2*, *smg-6*, and *eri-1*, however, reduced expression or the immediate visible effect of RNAi on the nematodes following *in vitro* dsRNA soaking was transient, and the phenomenon has been described as “Recovery”. Recovery from RNAi treatment or the associated stress of soaking is measured by a restoration of transcript abundance of target genes or a reversion to normal behavior and/or subsequent regaining of the nematodes ability to infect and develop normally in a host plant. An example was demonstrated for *smg-2*, *smg-6*, and *eri-1* genes in this study, where despite the significant transcript reduction (*p* < 0.05) and the associated changes in behavior, the nematodes recovered enough to infect and develop in the tomato hosts resulting in no significant difference in infestation compared to the controls (*p* < 0.05). The reasons for the recovery phenomenon, which has also been demonstrated in *M. graminicola*, *M. incognita*, *P. thornei*, and *Pratylenchus coffeae* (Rosso et al., 2005; Joseph et al., 2012; Nsengimana et al., 2013; Tan et al., 2013), are not currently known. It is possible the initial drop in transcript level is immediately detected as hazardous by the nematode, which then acts quickly to increase gene expression (homeostasis). Observations following induction of RNAi of the *ego-1* gene of the J2s of *M. incognita* were unusual; the extreme lethargic and light-insensitive phenotypes akin to dead nematodes were not matched by a reduction in gene expression, and yet the nematodes recovered enough somehow when they were placed on a host plant and were able to migrate and develop normally as they would without any RNAi treatment. It is plausible that, for this gene, a small immeasurable reduction in transcripts only transiently affected the nematodes leading to the observable phenotypes recorded. The conclusion from these observations is that it may not be enough to select possible RNAi targets for nematode control *via* RNAi based on just the initial gene knockdown or immediate defects in the nematode (behavior) before infection of host plants, and that especially for genes whose functions are as essential to nematodes as those involved in the RNAi pathways, a thorough investigation of the longer term effects of RNAi using HIGS is needed.

For HIGS of PPNs to be effective, the cell contents of transgenic plants from which the nematodes derive nourishment should include dsRNA of the target gene or the equivalent plant-processed siRNAs. Confirmation of expression of the target nematode genes in the transgenic plants provided evidence that the hairpins were transcribed. Any reduction of infection levels or impaired development of nematodes feeding on these plants, could, therefore, be attributed to the plant-processed dsRNA and/or siRNAs of the target genes. In particular, the plant-processed hairpin RNA of the microprocessor *drsh-1* induced the most reduction in nematode infestation. There was a weak negative correlation (*R* = -0.0889, *R*^2^ = 0.0079, *p* = 0.85) between the average percentage reduction in infection by the long dsRNA-treated J2s and that of J2s for which RNAi was induced by plant-derived dsRNA and/or siRNA. In general, though, it appears the *in vitro*-treated nematodes were less infective than those feeding on transgenic plants; the mean percent reduction in galls by the former was higher than the mean reduction in infectivity on most of the transgenic lines harboring hairpin RNA of the same gene. The difference in the infectivity of the two sets of nematodes could be attributed to the different dsRNA triggers they may have been presented with; the HIGS nematodes possibly were presented with additional plant-processed primary and secondary siRNAs. It can be argued that the pattern of the results was somewhat expected because the fitness of the *in vitro*-treated nematodes was reduced before they infected the host. This point, on the other hand, strengthens the effectiveness of HIGS where the level of nematode infestation can be drastically reduced after fit nematodes succeed in initially infecting the transgenic plants. It is reasonable to expect that HIGS of carefully selected target genes will therefore reduce nematode infectivity more than when the gene is knocked down *via in vitro* soaking because in the former case, the nematodes may ingest a mixture of dsRNA and plant-processed primary and possibly secondary siRNAs which may be produced through the siRNA amplification system of plants (Chen et al., 2010; Fosu-Nyarko et al., 2017). Nonetheless, the efficiency of HIGS may depend on all the factors that affect the expression of transgenes including copy number, site of insertion of different T-DNAs, and DNA methylation (Hobbs et al., 1990; Schubert et al., 2004). These factors may selectively affect different transgenic lines and could explain the differences in effectiveness of the different lines harboring the same hairpin in reducing nematode infectivity.

Nonetheless, a significant outcome of this research was that HIGS of all the six genes studied resulted in a significant level of reduction in nematode infectivity further confirming that genetic engineering of transgenic plants carrying hairpins of genes involved in the RNAi pathways can be used successfully to control *M. incognita* (Yadav et al., 2006; Fosu-Nyarko and Jones, 2015). The overwhelming evidence is that for some of these genes, although their importance to the nematode is underlined by the difficulty in reducing expression levels via exogenous RNAi, they could be applied *via* HIGS to control the nematodes. Such application for generating resistant commercial crops would involve careful selection of the transgene sequence to ensure there are absolutely no off-targets, either for the host plant or non-target organisms. This process will then be followed by pre-screening by soaking nematodes in homologous dsRNAs after conditions are optimized and the potential for recovery of the nematodes from RNAi effects are known or recalcitrance, if any, are noted. It is only really possible to ensure there are no off-target effects once full genomic sequences are available for the crop plant of interest, and for other non-nematode species that might encounter that crop.

## Data Availability Statement

Publicly available datasets were analyzed in this study. These data can be found here: https://www.ncbi.nlm.nih.gov/. Accession numbers of sequences are included in the Supplementary Material.

## Author Contributions

JF-N and MJ designed the research. SI conducted the experiments and collected all the data. All authors contributed to the data analyses, interpretation, and writing of the manuscript.

## Conflict of Interest

The authors declare that the research was conducted in the absence of any commercial or financial relationships that could be construed as a potential conflict of interest.

## Footnotes

1 www.wormbase.org

2 https://arasystem.com/aracons
