# Supplementary material for: Attempt to Silence Genes of the RNAi Pathways of the Root-Knot Nematode, Meloidogyne incognita Results in Diverse Responses Including Increase and No Change in Expression of Some Genes
Source: Front Plant Sci. 2020 Mar 24;11:328. doi: 10.3389/fpls.2020.00328 (PMC7105803; doi:10.3389/fpls.2020.00328)
Supplement: Supplementary file 1 [file Data_Sheet_1.docx]

Supplementary Material

**Supplementary Method:** Assembly of hairpin RNA interference constructs.

The cloning vector, pCleaver (Supplementary Figure S1), constructed by Dr. John Fosu-Nyarko was used to assemble the hairpin of the target genes. The sense and antisense fragments of target genes were cloned sequentially on either side of the bean catalase gene intron (190 bp). The cassette was driven by the Cauliflower Mosaic Virus 35S promoter and the Nopaline Synthase, Nos, terminator sequence. For each target gene, the 5′− 3′ (sense) fragments were digested out of pDoubler (Supplementary Figure S2) using the restriction enzymes *XhoI* and *KpnI*, except for *xpo-1* and *gfp* for which *AfeI* was used with *KpnI* and ligated to pCleaver linearised with the same restriction enzymes. After *E. coli* transformation, the presence of the sense and antisense fragments in transformants were respectively confirmed using primer pairs S35S-R (5′-GATTGATGTGACATCTCCACTGA-3′) and SIntron (5′-TCATCATC ATCATAGACACACGA-3′), ASIntron (5′-TCGTGTGTCTATGATGATGATGA-3′) and ASNosA (5′-CATCTCATAAATAACGTCATGCATT-3’). For the *gfp* cassette, the antisense restriction sites *AvrII* and *AscI* were used. The promoter, sense of target sequence, intron, antisense of target sequence and the terminator cassette was then digested with the restriction enzyme *NotI* and ligated to *NotI*-linearised and dephosphorylated binary vector pART27 (Wesley et al., 2001). After sequencing to confirm integrity of each construct, plasmid DNA of the vectors were then used to transform *Agrobacterium tumefaciens* strain GV3101 for plant transformations.

**
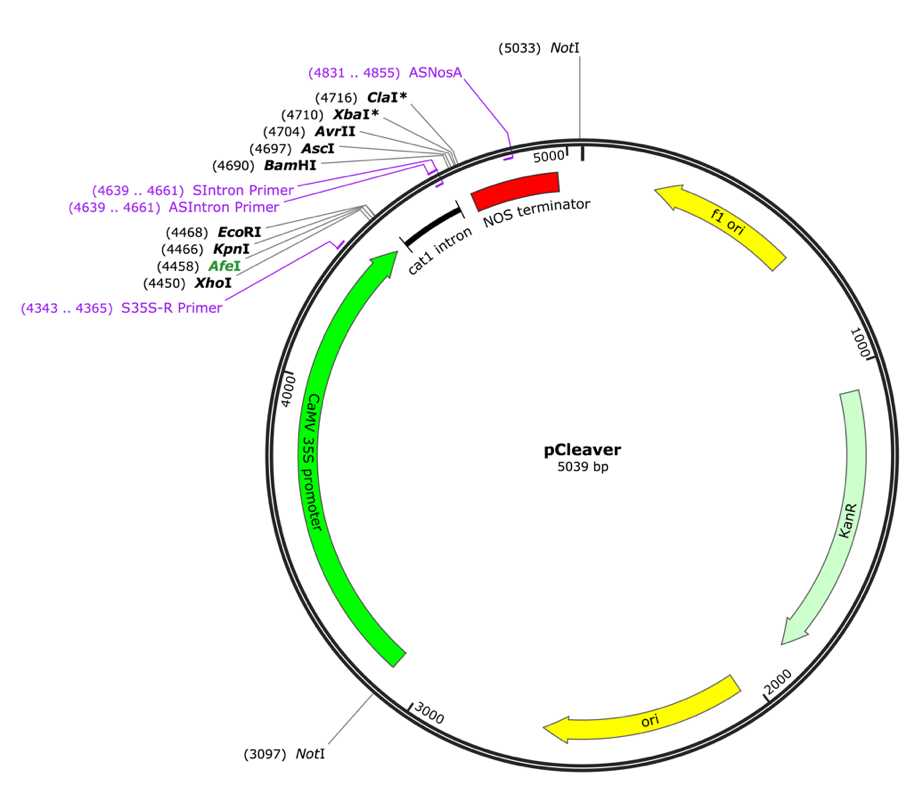
**

**Supplementary Figure S1:** Map of the hairpin cloning vector, pCleaver.

**
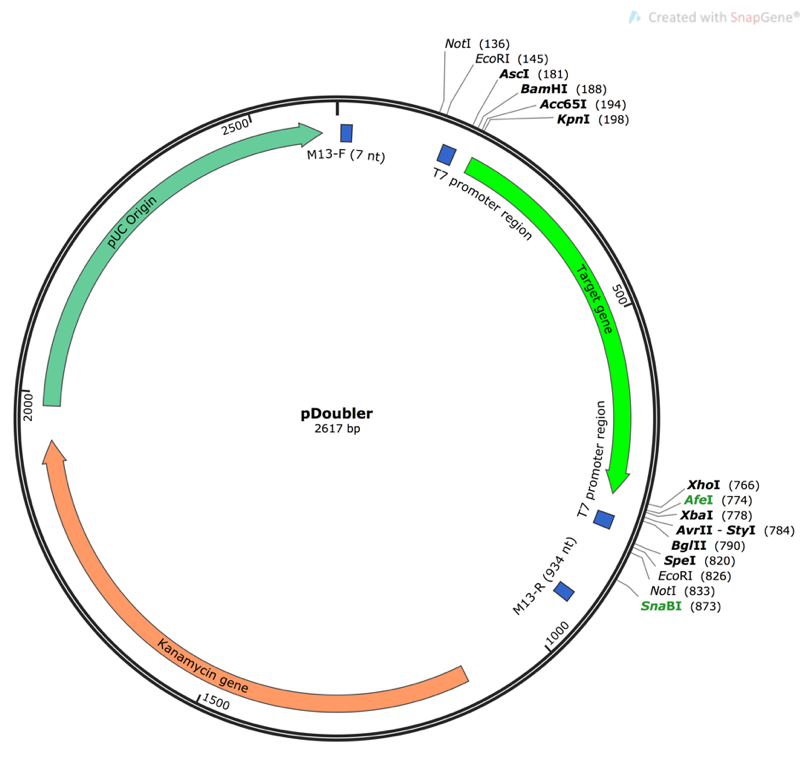
**

**Supplementary Figure S2:** Map of the transcription vector, pDoubler.
