# Supplementary material for: Attempt to Silence Genes of the RNAi Pathways of the Root-Knot Nematode, Meloidogyne incognita Results in Diverse Responses Including Increase and No Change in Expression of Some Genes
Source: Front Plant Sci. 2020 Mar 24;11:328. doi: 10.3389/fpls.2020.00328 (PMC7105803; doi:10.3389/fpls.2020.00328)
Supplement: Supplementary file 2 [file Data_Sheet_2.docx]

Supplementary Material

**
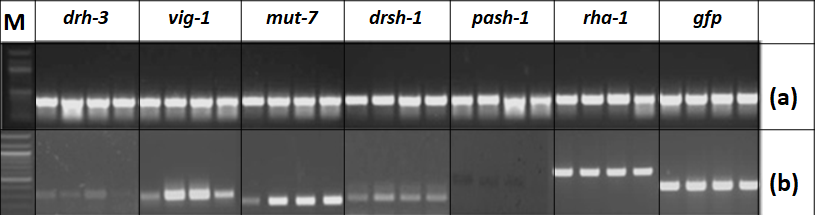
**

**Supplementary Figure S3:** Molecular analyses of transgenic Arabidopsis lines. (a) Amplicons of the *nptII* gene obtained from genomic DNA of four independent transgenic lines for each nematode hairpin construct. (b) Amplicons of nematode transgenes obtained from cDNA of four independent transgenic lines. M = 100bp DNA ladder. (Figure is a composite gel image).
