# Supplementary material for: Attempt to Silence Genes of the RNAi Pathways of the Root-Knot Nematode, Meloidogyne incognita Results in Diverse Responses Including Increase and No Change in Expression of Some Genes
Source: Front Plant Sci. 2020 Mar 24;11:328. doi: 10.3389/fpls.2020.00328 (PMC7105803; doi:10.3389/fpls.2020.00328)
Supplement: Supplementary file 3 [file Data_Sheet_3.docx]

Supplementary Material

**Supplementary Figure S4:** Mean number of galls per gram of dry root weight induced by *M. incognita* J2s on wild-type Col 0 (Wt) and nine T2 transgenic lines of Arabidopsis expressing *gfp* hairpin (Gfp-1 to 9). There were no significant differences (p<0.05) between the means of the Gfp lines (n = 7-10) and the means of the replicates (n=16) of the wild-type plants.
