# Supplementary material for: Attempt to Silence Genes of the RNAi Pathways of the Root-Knot Nematode, Meloidogyne incognita Results in Diverse Responses Including Increase and No Change in Expression of Some Genes
Source: Front Plant Sci. 2020 Mar 24;11:328. doi: 10.3389/fpls.2020.00328 (PMC7105803; doi:10.3389/fpls.2020.00328)
Supplement: Supplementary file 4 [file Table_1.docx]

Supplementary Material

**Supplementary Table S1:** Genes with roles in the small RNA pathway of *M. incognita* used for the study, Genbank accession numbers and target regions from where primers were designed.

| **Functional group of gene** | **Protein domains** | **Gene name**  **(Accession No.)** | **Primer used for target sequences**  **(5’-3’)** | **Amplicon size (bp)** |
| --- | --- | --- | --- | --- |
| Transport proteins | ENTH-Epsin | *rsd-3*  (CABB01006346) | F-ATTTGCCCCTTCATCTTTTCCTC  R-ACTGAAACTGAACAAAAAGTTCGTG | 314 |
|  | XPO1 | *xpo-1*  (CABB01004119) | F-TAAGATCGTCAAGCAAGAATG  R-TGAGATCTATCAGATTGTTCT C | 323 |
|  | CAS_CSE | *xpo-2*  (CABB01000462) | F-ATCTAGCTGCGCCAATAAC  R- TGATGTTGCTGAATTTAAACC | 587 |
| Dicer complex | Helicase C Terminal domain | *drh-1*  (CABB01006008) | F-GTCAAGCAAGTCGCGAAG  R- CCCTTCTTTGAACTCTAGCA | 192 |
|  | Ribonuclease III | *drsh-1*  (CABB01000477) | F-TAGAATTTCTTGGAGATGCTGTTG  R- ATCACATTGTTCAAGACCAGAATC | 280 |
|  | - | *pash-1*  (CABB01004277) | F-TCAGCATAA ACCTACTCGTG  R-TCGGCTTTG AATCAATTTCAAG | 539 |
| RISC protein | HABP4_PAI-RBP1 | *vig-1*  (CABB01000081) | F-TTTCGGTCGCGCCGTTTTG  R-AATCTTCTCATCAGCTCCTTCGCC | 205 |
| Amplification proteins | P loop AAA12 | *smg-2*  (CABB01008394) | F-ATGCCATAACGATTGTCTACTC  R- CAAATTCTGGCCTCGGAC | 301 |
|  | PIN | *smg-6*  (CABB01000011) | F-GTATCAATTTATGTAGACGC  R- TGGAAATGTTCGGACAGG | 131 |
|  | RdRp | *ego-1*  (CABB01000449) | F-CGAACTCAAGAACCTTTTTTCCG  R-CTGCTCGTTGATGTTTAAGTGC | 342 |
| RNAi inhibitors | DEDDh 3’-5’ exonuclease domain | *eri-1*  (CABB01001883) | F-GTGATTGATTTTGAATGTAGCTGTG  R- AAGCATCATCCATTCCACAATGTTC | 473 |
|  | Yeats | *gfl-1*  (CABB01000795) | F-AACAGGTTTCTCGTTGACGTCAG  R- TTCCTCAGAAAAACAGCAAAGGG | 281 |
| Nuclear RNAi proteins | Helicase C Terminal domain | *rha-1*  (CABB01000079) | F-TGGGTTTAAGAGGAATTTCTC  R- CATAAACAACATCATCAATTG | 669 |
|  | SET | *mes-2*  (CABB01002321) | F-CCACATTTTATTGACTGTTGG  R- TGCGAATGAGGATATCGAGA | 258 |
|  | WD40 | *mes-6* (CABB010000967) | F-TACAGTTGAAAAACTCATACCCC  R- AACTTTCAGGCCACACTC | 171 |
|  | Exoribonuclease domain | *mut-7*  (CABB01000055) | F-TCCAACACCAATTTT CTCAGCC  R- TCTGAGCAAGGCCTTTCC | 187 |
|  | DMAP1 | *ekl-4*  (CABB01002952) | F-TCGTTGGCCTGAATATAGAC  R- GATTTGACAATTGGTCAAGC | 245 |
| Argonautes | PIWI | *csr-1*  (CABB01000355) | F-CTGAAGTTCATCTTGAGTCA  R- TTGGACTCAACTACGTTC | 674 |
|  | PIWI | *ppw-2*  (CABB01001343) | F-AACCGAAGTCGTCACACA  R- CTTTGCCGAAATTCCATGTTC | 661 |
|  | PIWI | *2242*  (CABB01002242) | F-GCAGCATAGTGATGTCTAG  R- GCCAACGCTCTTTA AGG | 696 |
|  | - | *gfp*  (M62653.1) | F-TAACTCGAGTCTAGATTCACTGGAGTT  R- TACGGTACCGGATCCTAATGATCAGC | 524 |
|  | Cuticle collagen-Collagen triple helix | *rol-6*  (CABB01000004) | F-GGCTATTGCTTTTAGCGGAGC  R- TGCCATGATCTCCCGACTTCC | 609 |
|  | Ef-hand calcium binding motif | *pat-10*  (CABB01000228) | F-TTCAATCAGTCTCTCCAGCC  R- AATTCGACGCAGACGGCAG | 303 |
|  | - | NptII-F | F-TGCTCCTGCCGAGAAAGTAT  R- AATATCACGGGTAGCCAACG | 364 |
