# Supplementary material for: Attempt to Silence Genes of the RNAi Pathways of the Root-Knot Nematode, Meloidogyne incognita Results in Diverse Responses Including Increase and No Change in Expression of Some Genes
Source: Front Plant Sci. 2020 Mar 24;11:328. doi: 10.3389/fpls.2020.00328 (PMC7105803; doi:10.3389/fpls.2020.00328)
Supplement: Supplementary file 5 [file Table_2.docx]

Supplementary Material

**Supplementary Table S2**: Primers used to estimate fold change of target gene expression in *M. incognita* J2s after soaking in dsRNA.

| **Gene name** | **Primer name and sequence 5’-3’** | **Target amplicon**  **size (bp)** |
| --- | --- | --- |
| *actin* | qMiActin-F GTGCCATCCAAGCCGTTCTT  qMiActin-R ATTGCGTGTGGCGAAGCATA | 121 |
| *rsd-3* | qMiRsd3-F ATCAACACATTTGTAACTTTCCAAAGCC  qMiRsd3-R TGGAAGCGAACGAGTTATTTCTACAG | 79 |
| *xpo-1* | qMiXpo1-F GCACAACTCATTGGCACTTT  qMiXpo1-R CTTGACCTCCAGCTGTTCTT | 90 |
| *xpo-2* | qMiXpo2-F TTGTGGACGTTGGCAGATAG  qMiXpo2-R CAACGATTTATGGGCACAGTTT | 103 |
| *drh-1* | qMiDrh1-F AAGTCGCGAAGAAGGAGGTC  qMiDrh1-R CAGCAACCGTTGTTGCACA | 101 |
| *drsh-1* | qMiDrsh1-F CAAGTGAATATCTTTACAAACAATTTCC  qMiDrsh1-R CCTGTGGATAACCAAATATTTAACC | 140 |
| *pash-1* | qMiPash1-F CGAAGGTTTTGGCCCTGGTA  qMiPash1-R AGCAATTCCGTCAACATCAAACTC | 103 |
| *vig-1* | qMiVig1-F GAAGAACTGAGATGTTGCTCCTGA  qMiVig1-R CCGAACGGCTTCCAAAGAC | 100 |
| *ego-1* | qMiEgo1-F TTCCGACTTGTTCTACGTGCAG  qMiEgo1-R ACTTGGCCGTATTGTAATGAATCAG | 140 |
| *smg-2* | qMiSmg2-F GGAAAGGATGACAAAACAGGATGC  qMiSmg2-R GCAAATTCTGGCCTCGGACAATC | 110 |
| *smg-6* | qMiSmg6-F GTGGACCACATGCAGGATTA  qMiSmg6-R GTACGGAAAGCGCTCGAATA | 111 |
| *eri-1* | qMiEri1-F GTGATTGATTTTGAATGTAGCTGTGAAG  qMiEri1-R TTACAAGATTGCACGCTAATCATGAC | 98 |
| *gfl-1* | qMiGfl1-F ATCCTGTCTCTGACAACTCGAATG  qMiGfl1-R AGTTCAATTTCGTCTACACGAGTCTTATC | 83 |
| *mut-7* | qMiMut7-F TGTGCATCAAGAGCTGCATATCT  qMiMut7-R CGAAGAGTATTAGGCAAGCCGTT | 104 |
| *mes-2* | qMiMes2-F TCGCATTCATCAGAACATCCA  qMiMes2-R CTTGCCAACACAAAGGAGATTG | 100 |
| *mes-6* | qMiMes6-F GGCTTCCCAATTGCCAAACTT  qMiMes6-R ACTTTCAGGCCACACTCATTATGTT | 113 |
| *rha-1* | qMiRha1-F ATTACATTGGCTGAACGTGTTG  qMiRha1-R GCACCATATTGTCTTGGTGTTATT | 104 |
| *ekl-4* | qMiEkl4-F TACACAATCGGAACCTTTAGCA  qMiEkl4-R CCTTGCCTGTTCTTCTGTTCTA | 97 |
| *ppw-2* | qMiPpw2-F GTACTTCGTCAACTCCACGTTAT  qMiPpw2-R TTGATGTCCAAAGCACAAACTATG | 101 |
| *csr-1* | qMiCsr1-F ATGAGAGTAGCAAAGTCCGTTC  qMiCsr1-R GGAACTGGTAAACCTGTTGAGTA | 99 |
| *2242* | qMi2242-F TTCCAACCAAAGCAGAAATTGA  qMi2242-R AAGAATCGCCAACGCTCT | 90 |
